# Functional and Cognitive Occupational Therapy (FaC_o_T) Improves Self-Efficacy and Behavioral–Emotional Status of Individuals with Mild Stroke; Analysis of Secondary Outcomes

**DOI:** 10.3390/ijerph20065052

**Published:** 2023-03-13

**Authors:** Tal Adamit, Jeffrey Shames, Debbie Rand

**Affiliations:** 1 Department of Occupational Therapy, Sackler Faculty of Medicine, Tel Aviv University, Tel-Aviv 6997801, Israel; 2 Maccabi Health-Care Services, Tel-Aviv 6812509, Israel

**Keywords:** self-efficacy, mood, rehabilitation, participation, mild stroke

## Abstract

Background: Mild stroke is characterized by subtle impairments, such as low self-efficacy and emotional and behavioral symptoms, which restrict daily living. Functional and Cognitive Occupational Therapy (FaC_o_T) is a novel intervention, developed for individuals with mild stroke. Objectives: To examine the effectiveness of FaC_o_T compared to a control group to improve self-efficacy, behavior, and emotional status (secondary outcome measures). Material and Methods: Community-dwelling individuals with mild stroke participated in a single-blind randomized controlled trial with assessments at pre, post, and 3-month follow-up. FaC_o_T included 10 weekly individual sessions practicing cognitive and behavioral strategies. The control group received standard care. The New General Self-Efficacy Scale assessed self-efficacy; the Geriatric Depression Scale assessed depressive symptoms; the Dysexecutive Questionnaire assessed behavior and emotional status; and the ‘perception of self’ subscale from the Reintegration to Normal Living Index assessed participation. Results: Sixty-six participants were randomized to FaC_o_T (n = 33, mean (SD) age 64.6 (8.2)) and to the control (n = 33, age 64.4 (10.8)). Self-efficacy, depression, behavior, and emotional status improved significantly over time in the FaC_o_T group compared with the control, with small to large effect size values. Conclusion: The efficacy of FaC_o_T was established. FaC_o_T should be considered for community-dwelling individuals with mild stroke.

## 1. Introduction

Mild stroke is often overlooked, since individuals are independent in mobility and self-care and experience minimal neurological deficits [1]. However, individuals with mild stroke often experience difficulties in returning to their premorbid instrumental activities of daily living (IADL, such cooking or shopping), leisure activities, and work [1,2]. In addition, they typically experience mood disorders (such as depression and anxiety) as well as personality and behavioral changes [3,4], which might prevent them from returning to their life before the stroke [5]. These changes may include emotional instability, hypersensitivity, difficulty in expressing emotions, aggression, irritability, or apathy [6]. Individuals with mild stroke have also reported feelings of ‘loss of control’ and ‘chaos’, which may lead to changes in their perception of ‘self’ [7]. These perceptions, which are often not apparent to others, may explain their restricted participation and low self-reported quality of life [8,9].

Additionally, individuals with mild stroke may experience a gap between their actual and perceived ability to perform previous or new meaningful occupations [6], attributed to insecurity and low self-efficacy. Self-efficacy, defined as the individual’s belief in their ability to perform a skill or task as well as belief in their behavior [10], is one of the core concepts of Bandura’s Social Cognitive Theory. Self-efficacy influences how people feel, think, motivate themselves, and behave in relation to their health [11]. Self-efficacy is also related to goal setting, making choices, investing effort and resources to achieve goals, as well as resilience to failures, perceived difficulties, and stressful situations [12]. Individuals with higher self-efficacy have a stronger commitment to accomplish their goals and have a better sense of daily occupational performance and wellbeing [13,14] as opposed to individuals with lower self-efficacy.

The research regarding self-efficacy in stroke rehabilitation has addressed mainly two areas: self-care self-efficacy and fall self-efficacy (also termed balance confidence) and its association [15,16,17,18] with activities of daily Living (ADL), mood, and quality of life [19,20,21,22,23]. Self-efficacy has seldom been the focus of research, and very few intervention programs have been developed and researched specifically to enhance self-efficacy of individuals with stroke [9]. Improvements in mobility, ADL, depression, and quality of life were demonstrated following interventions, but self-efficacy was not positively affected [19,20,21,22,23]. Self-management programs have been used for individuals with different health conditions, including stroke, to affect behavior, influence the ability to cope, and adapt to and manage a health condition, but research regarding the efficacy of these programs is relativity rare [24]. The ‘Bridges self-management program’, for example, developed by Jones et al. [23] for individuals post stroke, includes one to six individual sessions using goal setting and strategies to promote specific behavior and problem solving to improve self-efficacy, functional activity, participation, and mood. Positive findings were seen in their preliminary study [23,25] and feasibility RCT [26]; participants exhibited increased self-efficacy and sense of control and improved functional activity and mood; however, only 12 individuals participated in the intervention.

The Functional and Cognitive Occupational Therapy (FaC_o_T) intervention [27] was developed according to the Template for Intervention Description and Replication (TI DieR) framework [28] (see Table S1) specifically to address the unique needs and consequences of mild stroke [29], in order to overcome functional barriers and to promote the health and wellbeing of patients [30,31]. The primary goal of FaCoT is to improve the daily performance and satisfaction with performance of individuals with mild stroke. Our previous publication [27] demonstrated statistically and clinically significant improvements with large effect sizes in the primary outcome measures—daily performance and satisfaction with performance, as well as participation—compared to standard care. In this paper, we aim to assess changes in self-efficacy, behavior, emotional status, and self-perception (our secondary outcome measures). The current study will help us to understand the improvements in daily performance and satisfaction with performance of the participants, reported earlier. We hypothesized that individuals who received FaC_o_T would improve their self-efficacy, behavior and emotional status, and self-perception compared with individuals who received standard care as assessed post intervention and at a 3-month follow-up.

## 2. Materials and Methods

### 2.1. Design

This is a single-blind, randomized controlled trial (NCT02925637), which was approved by the Helsinki (BB0010/16) and University ethics committees. Assessments were conducted pre (T1), post the 10-week FaC_o_T intervention or control (T2), and at a 3-month follow-up by experienced occupational therapist assessors who were blind to group allocation. The study and reporting are guided by the Consolidated Standards for Reporting Trials (CONSORT) guidelines [32].

### 2.2. Participants

Community-dwelling adults (age > 18 years) who sustained a mild stroke in the last 3 years were recruited for the study after signing a consent form. Mild stroke was determined by less than 5 points on the National Institutes of Health Stroke Scale (NIHSS) [33] and independence in basic activities of daily living (BADL). Individuals had to be able to understand, speak, and read the language, report having some functional and occupational difficulties since the stroke, and not have other neurological or psychiatric conditions. The sample size was calculated in G-Power analyses for *F*-test ANOVA repeated measures with 80% power and a significance level of 0.05 based on the primary outcome measure, the Canadian Occupational Performance Measure (COPM) [34]; 33 participants were recruited per group after accounting for a 15% dropout.

### 2.3. Randomization

Potential participants were invited to the assessment session (T1). Since this intervention is a functional–cognitive intervention, which might be impacted by the participant’s cognitive status, participants found eligible were stratified by cognitive status (by a Montreal Cognitive Assessment (MoCA) [35] score ≤22 points or ≥23 points) and then randomly assigned to either the FaC_o_T or the control group (ratio 1:1).

### 2.4. Intervention

FaC_o_T includes 10 weekly 1 h individualized sessions, led by an experienced occupational therapist (OT). It entails task analysis of the participant’s personal functional goals, defined as a goal to achieve a specific activity (such as preparing dinner or participating in social activities) that was identified using the COPM. Then, cognitive strategies of ‘initiation’, ’inhibition’, ‘planning’, and ‘decision making’ were taught and practiced in the first half of the session in different everyday scenarios. Then, behavioral strategies, i.e., ‘self-perception’, ’situation interpretation’, and ‘future prediction’, were taught and practiced using two personas—a positive persona (with high self-efficacy) and a negative persona (with low self-efficacy) in different everyday scenarios. Between the weekly sessions, participants were encouraged to perform daily activities and report back (success logs).

In line with the previous article, we will now demonstrate how all four of Bandura’s [36] sources were incorporated into FaC_o_T sessions in order to enhance self-efficacy: ‘Mastery Experience’, ‘Vicarious Experience’, ‘Verbal Persuasion’, and ‘Physiological Feedback’. ‘Mastery Experiences’, which is considered the most important factor, was attained by providing the participants with a sense of success using strategies to overcome a specific difficulty in daily living (based on the task analysis). The experience of success was also highlighted in the intervention by success logs, which helped raise the participants’ awareness, even when occupational goals were only partially achieved (for example, the participant initiated ‘small talk’ with one of his employees, as part of his goal to improve his interpersonal communication). Modeling and ‘Vicarious Experience’ were achieved using everyday scenarios of ‘case studies’ who had a stroke and, similar to the participant, experienced difficulties in daily living. By utilizing cognitive and behavioral strategies, participants with the OT analyzed the case studies to help them deal with different situations by utilizing strategies, which can be then used in their own life. ‘Verbal Persuasion’ was achieved using positive therapeutic language and positive feedback throughout FaC_o_T. In addition, the participants’ personal abilities, efforts, and progress were emphasized, which facilitated hope and increased the participants’ self-efficacy. ’Physiological Feedback’ was integrated by psycho-education and uncovering hidden symptoms and linking them to their function post stroke. In addition, physiological and emotional symptoms such as fatigue, cognitive impairments, and low self-efficacy were brought to the participants’ awareness, and the impact of these on their daily living was highlighted. In addition, by analyzing the behavior and thoughts of two personas, the implications of the different points of view were easily understood. As the sessions progressed, the participants gradually transferred these strategies to their own feelings and emotions, and they became more aware of their consequences for their daily activity and wellbeing.

**Figure 1:**
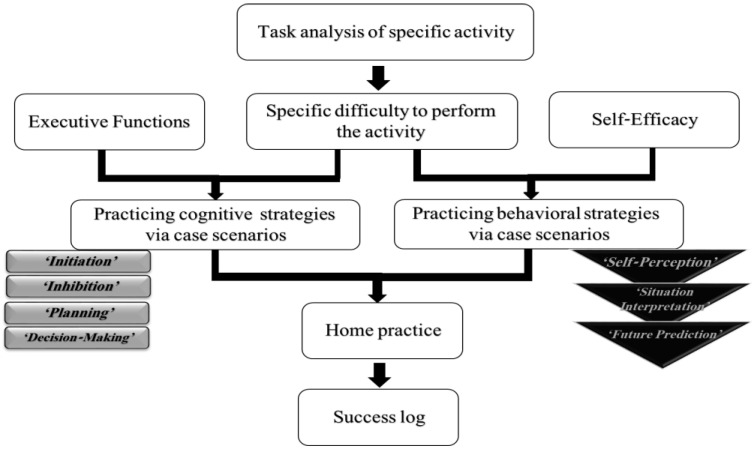
The process of FaC_o_T treatment sessions and the incorporation of the cognitive strategies (light gray rectangles) and behavioral strategies (black triangles). A task analysis of specific activities revealed the specific difficulty of the daily activities. Executive function deficits and low self-efficacy that explain this difficulty were analyzed, and then cognitive and behavioral strategies were used to overcome the difficulty. Participants practiced the use of strategies and were encouraged to perform daily activities at home. During the next session, participants shared their experiences, feelings, and emotions from the previous week.

Following each session, the OT filled in a fidelity checklist and kept a log of the participants’ comments and reactions. (See Figure 1 for a description of the FaC_o_T session process and the incorporation of strategies).

The control group did not receive rehabilitation services at the time of the study, which is considered standard care for most cases following mild stroke. They did undergo a full cognitive, behavioral, and emotional assessment (the same as the FaC_o_T group).

### 2.5. Instruments

The New General Self-Efficacy Scale (NGSE) [37] assessed self-efficacy. This self-report questionnaire comprises eight items that are rated using a 5-point Likert scale, from 1 (strongly disagree) to 5 (strongly agree), for example, “I will be able to achieve most of the goals that I have set for myself”, or “I am confident that I can perform effectively on different tasks”. The total score ranges from 8 to 40 points; a higher score indicates higher self-efficacy. The Geriatric Depression Scale (GDS) [38] was used to assess depressive symptoms. This 15-item self-report questionnaire ranges from 0 to 15 points; a score of 6 or higher indicates having depressive symptoms after stroke [39]. The Dysexecutive Questionnaire (DEX) [40] was used to assess the behavioral, emotional, and cognitive aspects related to the dysexecutive syndrome. It includes 20 questions rated on a 5-point Likert scale and produces three subscale scores [41]; the behavioral (0–32 points) and emotional (0–12 points) scores are reported here. The Reintegration to Normal Living Index (RNLI) [42] was used to assess participation by 11 statements regarding reintegration to productive, social, and leisure activities, rated from 0 (disagree) to 10 (strongly agree). In addition to the RNLI total score (0–100 points), two subscales can be calculated: ‘Daily Living’ (0–80 points) and ‘Perception of Self’ (0–30 points) [42], which evaluate how individuals perceive their ability to generally deal with situations. The RNLI ‘Perception of Self’ score was used as an additional measure of self-efficacy; higher scores indicate high self-perception.

In addition, we collected demographic (age, gender, education, and premorbid function), stroke (date, side, and type of lesion as well as the stroke severity measured by NIHSS [33]), and independence in daily living information (total score of the Functional Independence Measure (FIM) [43]).

### 2.6. Data Analysis

All data were analyzed using SPSS version 26. Descriptive statistics (*t*-tests for independent samples or the chi-square test) were used to describe the groups and the dependent variables at T1, T2, and T3. Normality testing of the data was performed using the Shapiro–Wilk test (*p* > 0.05). Differences between groups pre-intervention were analyzed using *t*-tests for independent samples (continuous measures) or chi-square tests (for dichotomous measures). A repeated measures 2(groups)X 3(time) analysis of variance ANOVA was used to compare within- and between-group scores, as well as for the interaction effect. To correct for the degrees of freedom, Mauchly’s test of sphericity was used, and the Greenhouse–Geisser procedure was conducted. Partial eta squared (*ɳ_P_^2^*) was used to calculate the magnitude of the difference; 0.01, 0.06, and 0.14 values were considered small, medium, and large effect sizes, respectively [44]. To better understand the main effect of time, post-hoc pairwise comparisons with Bonferroni correction were performed. Group effects were interpreted by *t*-test for independent samples with Cohen’s *d*. Intention-to-treat analysis was used with the last observation carried over [45].

## 3. Results

**Figure 2:**
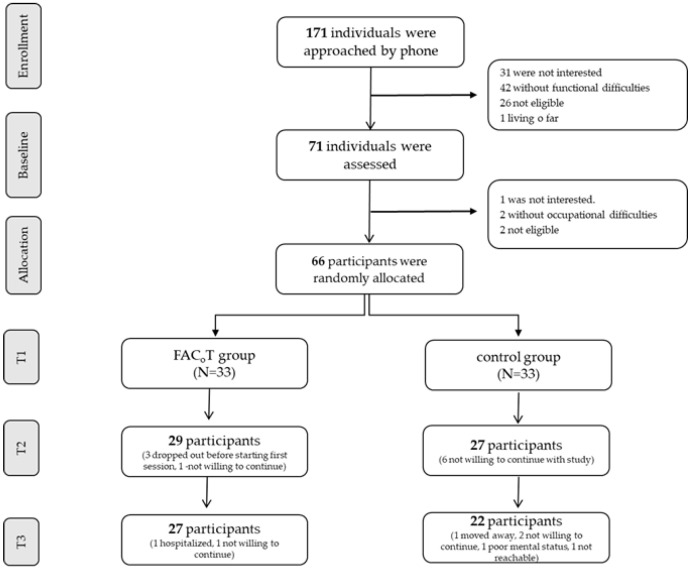
The study flow CONSORT diagram.

Individuals with mild stroke were recruited from lists from a community-based healthcare service between March 2017 and February 2020 and were randomly allocated to the FaC_o_T group ((*n* = 33, 33.3% women, mean (SD) age—64.6 (8.2)) or the control group ((*n* = 33, 45.4% women, mean (SD) age—64.4 (10.8)). See Figure 2 for the recruitment, allocation, and flow of participants. As shown in Table 1, most participants from both groups had a first ischemic subcortical mild stroke, and per inclusion criteria, they were independent in BADL. Participants from both groups identified four personal functional goals and reported low performance (FaC_o_T group mean (SD) 3.1 (1.3); control 3.7 (1.3) out of a maximum 10 points) and low satisfaction from their performance (FaC_o_T group 2.4 (1.3); control 3.1 (2.1) out of a maximum 10 points). In addition, their self-efficacy was somewhat low (FaC_o_T group 29.1 (7.7); control group 25.5 (9.5) out of a maximum 40 points), and 48.5% of the FaC_o_T group and 45.5% of the control group reported depressive symptoms. Groups were similar pre intervention (see Table 1).

### 3.1. Self-Efficacy

The Primary TimeXGroup effect with Greenhouse–Geisser correction was found for NGSE (*F*(1.5, 92.6) = 4.1, *p* < 0.03), with small to medium effect size values from T1–T2 (*ɳP^2^* = 0.02) and T1–T3 (*ɳ_P_^2^* = 0.06).

**Figure 3:**
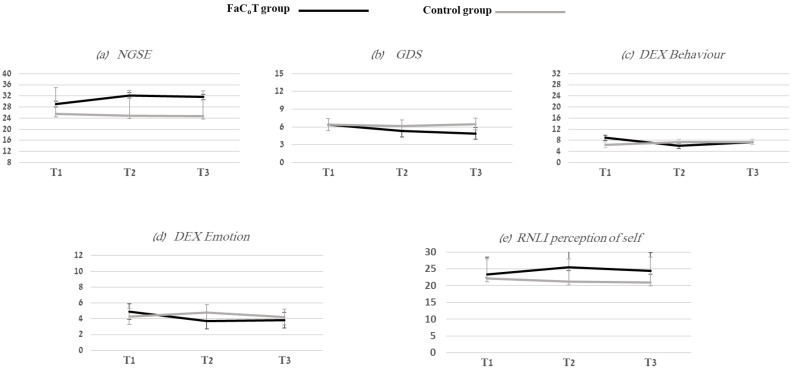
Five figures that show the mean (SD) of FaC_o_T (black lines) and control (gray lines) groups at T1, T2, and T3 for the following outcome measures: (**a**) NGSE—the New General Self-Efficacy Scale; (**b**) GDS—Geriatric Depression Scale; (**c**) DEX Behavior—Dysexecutive Questionnaire Behavior subscale; (**d**) DEX Emotion—Dysexecutive Questionnaire Emotion subscale; (**e**) RNL Perception of Self—Reintegration to Normal Living Index and the Perception of Self subscale.

A significant between-group effect (main effect) was also found for NGSE (*F* = 10.3, *p* < 0.002, *ɳ_P_^2^* = 0.14). The FaC_o_T group had higher mean (SD) scores compared to the control group at T2 (32.2 (6.4) compared to 24.8 (9.2); *t*(63) = 3.8, *p* < 0.001) and at T3 (31.6 (7.1), compared to 24.7 (9.1); *t*(63) = 3.4, *p* < 0.001)), with large effect sizes (Cohen’s *d* = 0.94; 0.84, at T2 and T3, respectively). (See Table 2 and Table 3, Figure 3). No within-subject effects were found.

### 3.2. Behavior and Emotional Status

The TimeXGroup effect was significant for GDS (*F*(2, 128) = 4.4, *p* < 0.01), DEX Behavior (*F*(2, 124) = 5.4, *p* < 0.006), and DEX Emotion (*F*(1.8, 109.4) = 4.3, *p* < 0.02), with medium to large effect size values from T1 to T2 (DEX Behavior *ɳP^2^* = 0.13; DEX Emotion *ɳP^2^* = 0.10), and T1 to T3 (GDS *ɳ_P_^2^* = 0.12; DEX Behavior *ɳ_P_^2^* = 0.06). (See Table 2 and Table 3, Figure 3). Mauchly’s test did not indicate any violation of sphericity for GDS (χ^2^(2) = 0.5, *p* < 0.79) or DEX Behavior (χ^2^(2) = 3.2, *p* < 0.21), except for DEX Emotion (χ^2^(2) = 8.7, *p* < 0.01). Greenhouse–Geisser correction to the degrees of freedom for DEX Emotion was completed.

Significant within-subject effects were found for GDS for both groups (*F*(2, 128) = 4.6, *p* < 0.01, *ɳ_P_^2^* = 0.07), with medium effect size values. Post-hoc analysis with a Bonferroni adjustment revealed that GDS significantly decreased from T1 to T3 (0.76 (95% CI, 0.08 to 1.44), *p* < 0.02). In the FaC_o_T group, 48.5% of individuals at T1 reported depressive symptoms, and only 33.3% reported these symptoms at T3. In the control group, 45.5% reported depressive symptoms at T1, and 48.5% at T3.

No between-subject effects were found.

### 3.3. RNLI Self-Perception

Significant between-group effects were found regarding the improvement of the RNLI self-perception scale (*F* = 5.4, *p* < 0.02, *ɳ_P_^2^* = 0.08). The FaC_o_T group had higher mean (SD) scores compared to the control group at T2 *25.4 (6.3), compared to 21.2 (6.8); t(64) = 2.6, *p* < 0.01) and at T3 (24.4 (5.4), compared to 20.9 (7.6); t(64) = 2.1, *p* < 0.04)), with a medium effect size (Cohen’s *d* = 0.64; 0.53, at T2 and T3, respectively). (See Table 2 and Table 3, Figure 3).

No within-subject main effect or TimeXGroup primary effect were found.

## 4. Discussion

This paper focused on evaluating the impact of FaC_o_T on self-efficacy, behavior, emotional status, and self-perception of individuals with mild stroke compared to a control group. Previously, we reported that participants who received FaC_o_T improved their performance and satisfaction in daily living [27]. Findings of this study also demonstrated improvement in self-efficacy, which is a person’s belief in their own ability and our secondary outcome measure. We can carefully suggest that these two aspects are related and had a mutual effect, as improvement in self-efficacy could have led to the improved occupational performance and satisfaction, and vice versa [27]. This positive change was also apparent at the three-month follow-up. These encouraging findings were achieved possibly because all four sources of Bandura’s theory [36] were incorporated into the FaC_o_T to increase self-efficacy, as suggested previously [46]. ‘Mastery Experience’, ‘Vicarious Experience’, ‘Verbal Persuasion’, and ‘Physiological Feedback’ were interpreted and adapted to promote self-efficacy and daily living and to achieve the participant’s personal goals. Stroke self-management programs have used different strategies and have focused on several domains, such as social support, communication, knowledge, goal setting, and lifestyle [47]. However, self-efficacy strategies to improve daily activity have rarely been used [48]. Previous stroke self-management programs have included small samples that were heterogeneous in terms of stroke severity and stage of recovery [49].

Significant improvements at T2 and T3 with medium to large effect size values for FaC_o_T compared with the control group were observed: a decrease in depressive symptoms (GDS) and an increase in the behavior and emotional status (DEX) for the FaC_o_T group. Post-stroke depression and emotional problems can negatively affect stroke recovery and rehabilitation [50,51,52,53] even after 6 months among individuals with mild to moderate stroke [54]; therefore, these findings are important. Aiming to explain these positive changes, we can suggest a few directions. Depression has a long-term negative effect on functional outcomes post stroke [55,56], and low functional ability may lead to an impact in depression, revealing a vicious cycle between the two constructs [57,58]. Therefore, possibly by improving activities of daily living and achieving their occupational goals (as we previously reported) [27], participants might have improved their emotional state. Executive function deficits are also associated with depression [59,60]; therefore, by teaching the use of cognitive strategies (for ‘initiation’, ‘inhibition’, ‘planning’, and ‘decision making’), participants might have felt more control and also improved their behavioral and emotional status.

The FaC_o_T group improved their self-perception to participate in daily activities at T2 as well at T3 compared with the control group. Individuals with (mild) stroke are often unaware of the precise impairments and the impact on their function and health [61,62]. The psycho-education aspect within FaC_o_T helped to uncover the participants’ hidden dysfunctions and link them to the stroke, making them aware of the consequences. Participants may have gained control over the situation as they became increasingly aware of both their abilities and limitations. Additionally, the use of the negative and positive personas within the sessions may have increased their awareness regarding how their self-perception may impact their daily living [63], leading to more improvement in the FaC_o_T participants.

Our study has several limitations. Our main limitation is that our control group did not receive an alternative intervention but rather received standard care. Therefore, although assessments pre, post, and at follow-up were administered, including defining occupational goals, the effects of meeting and talking with a supportive and compassionate therapist were not controlled for in this study. Participants were heterogeneous in terms of time since stroke, but most participants were in the chronic stage post stroke. Our 3-month follow-up period was relatively short; future research should include a longer follow-up period. We assessed the emotional and behavioral status and self-perception of individuals using subscales of acceptable assessments. Further research should also include full self-report questionnaires.

## 5. Conclusions

FaC_o_T has efficacy in enhancing the self-efficacy, emotional–behavioral status, and the self-perception of individuals with mild stroke compared with standard care. Therefore, the implementation of FaC_o_T as a community-based rehabilitation program should be considered for individuals with mild stroke, who usually do not receive formal rehabilitation.

## Figures and Tables

**Table 1:** Characteristics of the participants in the FaC_o_T and control groups at T1.

	FaC_o_T (*n* = 33)	Control Group (*n* = 33)	Differences between Groups
	Mean (SD), Min–Max	Mean (SD), Min–Max	
Age (years)	64.6 (8.2), 49–77	64.4 (10.8), 48–84	0.9 ^a^
Education (years)	12.1 (1.9), 8–16	12.9 (2.8), 6–20	0.2 ^a^
NIHSS (0–46) mean (SD)	1.2 (1.2), 0–4	1.7 (1.6), 0–6	0.2 ^a^
FIM (18–126) mean (SD)	118.8 (7.2), 98–126	117.2 (7.1), 96–126	0.4 ^a^
MoCA (0–30)	21.5 (3. 9), 11–29	21.8 (4.1), 14–28	0.8 ^a^
	N (%)	N (%)	
Sex female *n* (%)	11 (33.3)	15 (45.5)	0.3 ^b^
First stroke *n* (%)	20 (60.6)	19 (57.6)	0.8 ^b^
Stroke side R *n* (%)	13 (39.4)	14 (42.4)	0.7 ^b^
Type of stroke—ischemic/hemorrhage *n* (%)	32/0 (100/0)	31/2 (93.9/6.1)	0.2 ^b^
Lesion—cortical/subcortical *n* (%)	9/17 (27.3/51.5)	8/19 (24.2/57.6)	0.8 ^b^
Chronic stage *n* (%)	28 (84.8)	26 (78.8)	0.5 ^b^
Worked before stroke *n* (%)	20 (60.6)	13 (39.4)	0.1 ^b^
Returned to work since stroke *n* (%)	11 (33.3)	4 (12.1)	0.2 ^b^

SD—standard deviation, ^a^ *t*-test, ^b^ chi squared. Abbreviations: NIHSS, National Institutes of Health Stroke Scale; FIM, Functional Independence Measure; MoCA, Montreal Cognitive Assessment.

**Table 2:** The mean (SD) scores of the outcome measures of both groups at T1, T2, and T3.

	**FaC_o_T Group (*n* = 33)**			**Control Group (*n* = 33)**		
	**T1** **Mean (SD)**	**T2** **Mean (SD)**	**T3** **Mean (SD)**	**T1** **Mean (SD)**	**T2** **Mean (SD)**	**T3** **Mean (SD)**
NGSE (8–40)	29.1 (7.7)	32.2 (6.4)	31.6 (7.1)	25.5 (9.5)	24.8 (9.2)	24.7 (9.1)
GDS (0–15)	6.4 (3.3)	5.3 (3.8)	4.9 (4.1)	6.4 (4.0)	6.2 (3.6)	6.5 (3.9)
DEX Behavioral (0–32)	8.9 (6.4)	6.0 (5.3)	7.4 (6.5)	6.4 (5.9)	7.4 (5.7)	7.4 (6.5)
DEX Emotional (0–12)	4.9 (2.8)	3.7 (2.6)	3.8 (2.6)	4.3 (3.1)	4.8 (2.8)	4.2 (2.9)
RNLI self-perception (0–30)	23.3 (5.2)	25.4 (6.3)	24.4 (5.4)	22.2 (5.9)	21.2 (6.8)	20.9 (7.6)

Abbreviations: NGSE, New General Self-Efficacy scale (higher score indicates higher SE); GDS, Geriatric Depression Scale (lower score indicates fewer depressive symptoms); DEX, Dysexecutive Questionnaire Behavior and Emotion scale (lower score indicates fewer behavioral and emotional symptoms); RNLI, Reintegration to Normal Living Index self-perception scale (higher score indicates higher self-perception).

**Table 3:** A repeated measures ANOVA 2(groups) X 3(times), within and between groups, and the interaction effect and effect size.

	Interaction Effect (Time X Group)			Main Effect (Time)			Main Effect (Group)		
	*F*	*P*	*ɳ_P_^2^*	*F*	*P*	*ɳ_P_^2^*	*F*	*P*	*ɳ_P_^2^*
NGSE	4.1	0.03	0.06	1.4	0.24	0.02	10.3	0.002	0.14
GDS	4.4	0.01	0.07	4.6	0.01	0.07	0.9	0.33	0.01
DEX Behavioral	5.4	0.006	0.08	1.3	0.28	0.02	0.1	0.78	0.001
DEX Emotional	4.3	0.02	0.07	2.3	0.11	0.04	0.3	0.62	0.004
RNLI self-perception	2.2	0.12	0.03	0.4	0.67	0.01	5.4	0.02	0.08

*F*: F score; *p*: significance value; *ɳ_P_^2^*: partial eta squared; effect size: small—ɳ_P_^2^ = 0.01, medium—ɳ_P_^2^ = 0.06, large—ɳ_P_^2^ = 0.14. Abbreviations: NGSE, New General Self-Efficacy scale; GDS, Geriatric Depression Scale; DEX, Dysexecutive Questionnaire Behavior and Emotion scale; RNLI, Reintegration to Normal Living Index self-perception scale.

## Data Availability

The data presented in this study are available on request from the corresponding author. The data are not publicly available due to the immunity of patients’ medical information.

## Supplementary Materials

The following supporting information can be downloaded at: https://www.mdpi.com/article/10.3390/ijerph20065052/s1. Table S1, the Template for Intervention Description and Replication (TIDieR) framework describes the FaCoT intervention.
