# Supplementary material for: Functional and Cognitive Occupational Therapy (FaCoT) Improves Self-Efficacy and Behavioral–Emotional Status of Individuals with Mild Stroke; Analysis of Secondary Outcomes
Source: Int J Environ Res Public Health. 2023 Mar 13;20(6):5052. doi: 10.3390/ijerph20065052 (PMC10049253; doi:10.3390/ijerph20065052)
Supplement: Supplementary file 1 [file ijerph-20-05052-s001.zip › ijerph-2179693-supplementary.pdf]

The TIDieR (Template for Intervention Description and Replication) Checklist*:

Information to include when describing an intervention and the location of the information

Item number	Item
	**BRIEF NAME**
**1.**	FaC_oT
	**WHY**
**2.**	**<u>Rationale:</u>** FaC_oT was developed to focus on the unique characteristics of individuals with mild stroke, specifically experiencing cognitive, emotional -behavioral symptoms and experience difficulties in Instrumental Activities of Daily Living, leisure and work. Research evidence on the effectiveness of cognitive–functional occupational therapy interventions are limited<br>**<u>Goals:</u>** The primary goal of FaC_oT is to enhance occupational performance and satisfaction of individuals with mild stroke. Our secondary goal is to enhance self-efficacy, behavior, emotional status, and self-perception.
	**WHAT**
**3.**	***<u>Materials:</u>***<br>During the intervention sessions, the OT uses the following sheets, that are then given to the participant in a binder:<br>• Psycho-education sheets about hidden stroke symptoms and their consequences.<br>• Task analysis sheet regarding their personal functional goals<br>• Sheets with an explanation of different cognitive and behavioral strategies.<br>• Everyday scenarios sheets: Cognitive and behavioural strategies practiced in everyday scenarios.<br>• Success log sheets to record their progress, thoughts and feeling
**4.**	***<u>Procedures:</u>***<br>**<u>General information</u>**: Ten individual treatment sessions led by an experienced occupational therapist (OT).<br>**<u>First session includes</u>**:<br>• Psycho-education about mild stroke symptoms and their consequences

- Task analysis of the participant's personal functional goals that participants have defined by COPM that they aim to achieve (such as initiating dinner preparation or planning a social event).
- Teaching one cognitive strategy (initiation', 'inhibition', 'planning' or 'decision-making') that align to the participant's personal functional goals
- Teaching a behavioral strategy (self-perception, situation interpretation, future prediction)
  and the use of two personas - one positive (with high self-efficacy) and one negative (with low self-efficacy)

Sessions 2-10 included the following components:

- Practice of cognitive strategies in everyday scenarios
- Practice of behavioral strategies using the two personas in different everyday scenarios.
- In between the weekly sessions, participants are encouraged to perform daily activities and report back at the next meeting (including their progress, thoughts and feeling and documented in success logs).
- Positive therapeutic language and positive feedback was used during all sessions.
- The last session discusses all the concepts that were taught during the intervention.

WHO PROVIDED

5. The intervention was led by an Occupational Therapist who received training from the developer of the intervention (OT specialist). A manual of the intervention is also provided, so that the OT can replicate the intervention sessions.

HOW

6. Individualized sessions, face to face.

WHERE

7. Sessions took place in an outpatient community clinic or in the participant's home. Participants were recruited from health care services in community

WHEN and HOW MUCH

8. 10 1-hour weekly sessions

TAILORING

The same intervention was provided to all of the participants. The everyday functional scenarios and the strategies used & practiced were according to the specific goals and difficulties of the participant.

9. N/A

MODIFICATIONS

10.‡ N/A

HOW WELL

11. **Planned:** OT were trained to practice according to the protocol, and filled in a fidelity checklist following each session.

12.‡ **Actual:** The number of FaC_oT sessions was as planned and fidelity checklist was filled in for all participants. Only 3 participants dropped out before starting the first session

---
